# 3-Meth­oxy­methyl-16β,17β-epiestriol-16β,17β-diyl sulfate

**DOI:** 10.1107/S1600536811039213

**Published:** 2011-10-12

**Authors:** Jian-Jun Chen, Hai-Dong Wang, Cheng Yao

**Affiliations:** a Department of Applied Chemistry, College of Science, Nanjing University of Technology, No. 5 Xinmofan Road, Nanjing, Nanjing 210009, People’s Republic of China

## Abstract

The title compound, C_20_H_26_O_6_S, synthesized by the reaction of 3-*O*-meth­oxy­methyl-16β-epiestriol and sulfonyl­diimidazole, is composed of a 3-meth­oxy­methyl group connected *via* two O atoms to a 16,17-*O*-sulfuryl-16-epiestriol group. In the crystal, weak inter­molecular C—H⋯O hydrogen bonds link the mol­ecules into [001] chains.

## Related literature

We have used the title compound as a substrate for the production of F-18 16α-fluoro­estradiol *via* nucleophilic fluorination, see: Romer *et al.* (1996 ▶). Fluorine-18 16α-fluoro­estradiol is a valuable radiopharmaceutical for the investigation of the estrogen receptor status of primary and metastatic breast cancer, see: Lim *et al.* (1996 ▶); Romer *et al.* (1996 ▶). For bond-length data, see: Allen *et al.* (2002 ▶).
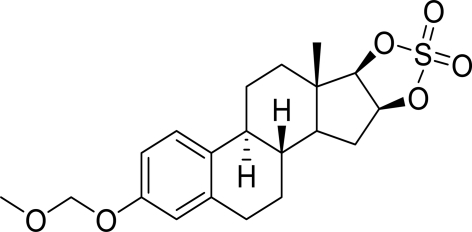


## Experimental

### 

#### Crystal data

C_20_H_26_O_6_S
*M* *_r_* = 394.47
Orthorhombic, 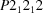

*a* = 10.296 (2) Å
*b* = 23.503 (5) Å
*c* = 7.9060 (16) Å
*V* = 1913.1 (7) Å^3^
*Z* = 4
Mo *K*α radiation
μ = 0.20 mm^−1^
*T* = 293 K
0.30 × 0.20 × 0.10 mm

#### Data collection

Enraf–Nonius CAD-4 diffractometer
Absorption correction: ψ scan (North *et al.*, 1968 ▶) *T*_min_ = 0.942, *T*_max_ = 0.980
3875 measured reflections
3530 independent reflections
2174 reflections with *I* > 2σ(*I*)
*R*_int_ = 0.060
3 standard reflections every 200 reflections  intensity decay: 1%

#### Refinement

*R*[*F* ^2^ > 2σ(*F* ^2^)] = 0.064
*wR*(*F* ^2^) = 0.182
*S* = 1.00
3530 reflections
244 parameters
H-atom parameters constrained
Δρ_max_ = 0.22 e Å^−3^
Δρ_min_ = −0.17 e Å^−3^
Absolute structure: Flack (1983 ▶), 1483 Friedel pairs
Flack parameter: 0.00 (18)

### 

Data collection: *CAD-4 Software* (Enraf–Nonius, 1989 ▶); cell refinement: *CAD-4 Software*; data reduction: *XCAD4* (Harms & Wocadlo,1995 ▶); program(s) used to solve structure: *SHELXS97* (Sheldrick, 2008 ▶); program(s) used to refine structure: *SHELXL97* (Sheldrick, 2008 ▶); molecular graphics: *SHELXTL* (Sheldrick, 2008 ▶); software used to prepare material for publication: *SHELXL97*.

## Supplementary Material

Crystal structure: contains datablock(s) global, I. DOI: 10.1107/S1600536811039213/bq2303sup1.cif

Structure factors: contains datablock(s) I. DOI: 10.1107/S1600536811039213/bq2303Isup2.hkl

Additional supplementary materials:  crystallographic information; 3D view; checkCIF report

## Figures and Tables

**Table 1:** Hydrogen-bond geometry (Å, °)

*D*—H⋯*A*	*D*—H	H⋯*A*	*D*⋯*A*	*D*—H⋯*A*
C20—H20*A*⋯O3^i^	0.96	2.50	3.338 (5)	146

Symmetry code: (i) 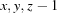.

**Fig. 1.**
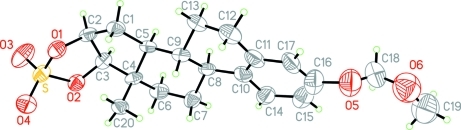
A view of the molecular structure of (I). Displacement ellipsoids are drawn at the 50% probability level.

**Fig. 2.**
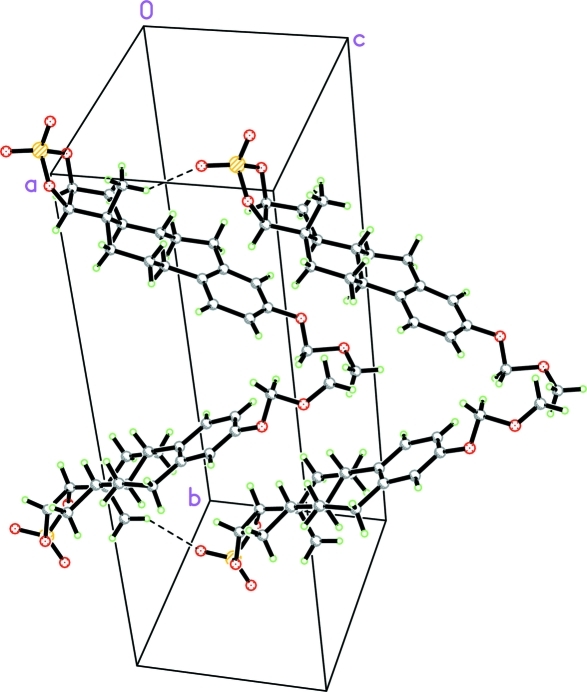
A packing diagram for (I). Dashed lines indicate an intermolecular C—H···O hydrogen bond.

## supplementary crystallographic information

### Comment

Fluorine-18 16alpha-fluoroestradiol has proven to be a valuable radiopharmaceuticalfor the investigation of the estrogen receptor status of primary and metastatic breast cancer. (Lim *et al.*,1996; Romer *et al.*,1996). We have prepared 3-methoxymethyl-16β,17β-epiestriol-O-cyclic sulfone and used it as a substrate for the production of F-18 16alpha-fluoroestradiol, *via* nucleophilic fluorination with fluoride ion. (Romer *et al.*,1996).

We report here the crystal structure of the titled compound, (I). The molecular strucutre of (I) is shown in Fig. 1. In this structure, ring A (S/O2/C3/C2/O1) and ring B (C1-C5) adopt twist conformation, the mean deviation from plane are 0.1171 Å and 0.1817 Å, respectively. Ring C (C4-C9) and ring D (C8-C13) are two twisty six-mermbered rings, the dihedral angles between the C7/C8/C9 and C5/C4/C6, C10/C11/C12 and C8/C9/C13 are 1.1 (1) ° and 43.3 (2) °, respectively. So it can be found that the ring C is a six-membered ring in its chair form. Ring E (C10/C11/C14-C17) is a planar six-mermbered ring and the mean deviation from plane is 0.0170 Å. In the crystal structure, intermolecular weak C—H···O hydrogen bond (Table 1) links the molecules to form a dimeric unit (Fig. 2), in which it may be effective in the stabilization of the structure.

### Experimental

A mixture of 3-*O*-Methoxymethyl-l6β-Epiestriol (0.9 mmol) and sodium hydride (3.5 mmol) in anhydrous THF (10 ml) was stirred for 15 min. Added a solution of sulfonyldiimidazole (0.9 mmol) in THF slowly, then stirred for 1 h, filtered through celite and washed with EtOAc to give pure compound (I) (m.p. 425–426 K).(Lim *et al.*,1996). Crystals of (I) suitable for X-ray diffraction were obtained by slow evaporation of an ethanol solution.

### Refinement

All H atoms were positioned geometrically, with C—H=0.98, 0.97, 0.96 and 0.93 Å for methine, methylene, methyl and aromatic H atoms, respectively, and constrained to ride on their parent atoms, with *U*_iso_(H)=*xU*_eq_(C), where *x*=1.5 for methyl H atoms and *x*=1.2 for all other H atoms.

### Crystal data

**Table:**

C_20_H_26_O_6_S	*D*_x_ = 1.370 Mg m^−^^3^
*M**_r_* = 394.47	Melting point = 425–426 K
Orthorhombic, *P*2_1_2_1_2	Mo *K*α radiation, λ = 0.71073 Å
Hall symbol: P22ab	Cell parameters from 25 reflections
*a* = 10.296 (2) Å	θ = 9–13°
*b* = 23.503 (5) Å	µ = 0.20 mm^−^^1^
*c* = 7.9060 (16) Å	*T* = 293 K
*V* = 1913.1 (7) Å^3^	Needle, colorless
*Z* = 4	0.30 × 0.20 × 0.10 mm
*F*(000) = 840	

### Data collection

**Table:**

Enraf–Nonius CAD-4 diffractometer	2174 reflections with *I* > 2σ(*I*)
Radiation source: fine-focus sealed tube	*R*_int_ = 0.060
graphite	θ_max_ = 25.4°, θ_min_ = 1.7°
ω/2θ scans	*h* = −12→12
Absorption correction: ψ scan (North *et al.*, 1968)	*k* = −28→0
*T*_min_ = 0.942, *T*_max_ = 0.980	*l* = −9→0
3875 measured reflections	3 standard reflections every 200 reflections
3530 independent reflections	intensity decay: 1%

### Refinement

**Table:**

Refinement on *F*^2^	Secondary atom site location: difference Fourier map
Least-squares matrix: full	Hydrogen site location: inferred from neighbouring sites
*R*[*F*^2^ > 2σ(*F*^2^)] = 0.064	H-atom parameters constrained
*wR*(*F*^2^) = 0.182	*w* = 1/[σ^2^(*F*_o_^2^) + (0.1*P*)^2^ + 0.2*P*] where *P* = (*F*_o_^2^ + 2*F*_c_^2^)/3
*S* = 1.00	(Δ/σ)_max_ < 0.001
3530 reflections	Δρ_max_ = 0.22 e Å^−^^3^
244 parameters	Δρ_min_ = −0.17 e Å^−^^3^
0 restraints	Absolute structure: Flack (1983), 1483 Friedel pairs
Primary atom site location: structure-invariant direct methods	Flack parameter: 0.00 (18)

### Special details

**Table:**

Geometry. All e.s.d.'s (except the e.s.d. in the dihedral angle between two l.s. planes) are estimated using the full covariance matrix. The cell e.s.d.'s are taken into account individually in the estimation of e.s.d.'s in distances, angles and torsion angles; correlations between e.s.d.'s in cell parameters are only used when they are defined by crystal symmetry. An approximate (isotropic) treatment of cell e.s.d.'s is used for estimating e.s.d.'s involving l.s. planes.
Refinement. Refinement of *F*^2^ against ALL reflections. The weighted *R*-factor *wR* and goodness of fit *S* are based on *F*^2^, conventional *R*-factors *R* are based on *F*, with *F* set to zero for negative *F*^2^. The threshold expression of *F*^2^ > σ(*F*^2^) is used only for calculating *R*-factors(gt) *etc*. and is not relevant to the choice of reflections for refinement. *R*-factors based on *F*^2^ are statistically about twice as large as those based on *F*, and *R*- factors based on ALL data will be even larger.

### Fractional atomic coordinates and isotropic or equivalent isotropic displacement parameters (Å^2^)

**Table:**

	*x*	*y*	*z*	*U*_iso_*/*U*_eq_	
S	0.28012 (13)	0.68651 (6)	0.51055 (15)	0.0796 (4)	
O1	0.3963 (3)	0.72326 (15)	0.4438 (4)	0.0812 (9)	
C1	0.4281 (4)	0.8080 (2)	0.2704 (6)	0.0746 (12)	
H1A	0.4782	0.7802	0.2075	0.089*	
H1B	0.4854	0.8383	0.3078	0.089*	
O2	0.1693 (3)	0.72213 (15)	0.4266 (4)	0.0780 (9)	
C2	0.3582 (4)	0.7803 (2)	0.4211 (6)	0.0719 (12)	
H2A	0.3711	0.8024	0.5248	0.086*	
O3	0.2689 (5)	0.6907 (2)	0.6849 (4)	0.1299 (16)	
C3	0.2124 (4)	0.7777 (2)	0.3724 (5)	0.0673 (11)	
H3B	0.1636	0.8076	0.4311	0.081*	
C4	0.2067 (4)	0.78685 (18)	0.1763 (5)	0.0635 (10)	
O4	0.2839 (4)	0.63112 (14)	0.4338 (5)	0.1001 (11)	
C5	0.3156 (4)	0.83170 (18)	0.1630 (6)	0.0606 (11)	
H5A	0.2838	0.8655	0.2228	0.073*	
O5	0.2892 (7)	0.9855 (3)	−0.7260 (7)	0.155 (2)	
C6	0.0819 (4)	0.8112 (2)	0.1126 (7)	0.0864 (15)	
H6A	0.0543	0.8420	0.1860	0.104*	
H6B	0.0151	0.7821	0.1142	0.104*	
O6	0.2753 (9)	1.0346 (3)	−0.9382 (11)	0.190 (3)	
C7	0.0990 (5)	0.8339 (3)	−0.0709 (7)	0.0858 (15)	
H7A	0.1155	0.8021	−0.1463	0.103*	
H7B	0.0191	0.8521	−0.1069	0.103*	
C8	0.2109 (5)	0.87663 (17)	−0.0848 (6)	0.0705 (11)	
H8A	0.1902	0.9082	−0.0084	0.085*	
C9	0.3391 (4)	0.85036 (18)	−0.0208 (6)	0.0670 (12)	
H9A	0.3592	0.8167	−0.0891	0.080*	
C10	0.2324 (6)	0.90293 (19)	−0.2624 (7)	0.0827 (14)	
C11	0.3484 (7)	0.9168 (2)	−0.3227 (8)	0.0948 (18)	
C12	0.4723 (6)	0.9051 (3)	−0.2252 (9)	0.1062 (19)	
H12A	0.5160	0.8727	−0.2753	0.127*	
H12B	0.5294	0.9377	−0.2354	0.127*	
C13	0.4483 (5)	0.8932 (2)	−0.0402 (7)	0.0838 (15)	
H13A	0.4254	0.9283	0.0173	0.101*	
H13B	0.5268	0.8784	0.0112	0.101*	
C14	0.1150 (8)	0.9149 (2)	−0.3591 (8)	0.104 (2)	
H14A	0.0322	0.9047	−0.3235	0.124*	
C15	0.1424 (10)	0.9461 (3)	−0.5261 (9)	0.126 (3)	
H15A	0.0727	0.9564	−0.5945	0.151*	
C16	0.2557 (12)	0.9581 (3)	−0.5736 (9)	0.120 (3)	
C17	0.3595 (9)	0.9437 (2)	−0.4803 (8)	0.110 (2)	
H17A	0.4420	0.9519	−0.5218	0.132*	
C18	0.2327 (12)	1.0332 (4)	−0.7645 (12)	0.168 (4)	
H18A	0.2656	1.0650	−0.6991	0.202*	
H18B	0.1389	1.0312	−0.7543	0.202*	
C19	0.1688 (12)	1.0446 (4)	−1.0307 (14)	0.196 (5)	
H19A	0.1908	1.0437	−1.1487	0.294*	
H19B	0.1342	1.0813	−1.0024	0.294*	
H19C	0.1049	1.0159	−1.0073	0.294*	
C20	0.2369 (5)	0.73070 (18)	0.0879 (5)	0.0754 (13)	
H20A	0.2351	0.7362	−0.0324	0.113*	
H20B	0.1732	0.7028	0.1191	0.113*	
H20C	0.3216	0.7178	0.1213	0.113*	

### Atomic displacement parameters (Å^2^)

**Table:**

	*U*^11^	*U*^22^	*U*^33^	*U*^12^	*U*^13^	*U*^23^
S	0.0769 (7)	0.1030 (9)	0.0588 (6)	−0.0014 (7)	−0.0024 (6)	−0.0010 (7)
O1	0.0633 (18)	0.096 (2)	0.084 (2)	0.0045 (17)	−0.0062 (16)	−0.0018 (19)
C1	0.054 (2)	0.077 (3)	0.093 (3)	−0.008 (2)	−0.003 (2)	−0.007 (3)
O2	0.0670 (18)	0.095 (2)	0.0718 (19)	−0.0097 (17)	−0.0016 (15)	0.0055 (18)
C2	0.068 (3)	0.079 (3)	0.069 (3)	0.007 (2)	−0.015 (2)	−0.025 (3)
O3	0.115 (3)	0.219 (5)	0.0553 (19)	0.021 (4)	0.010 (2)	−0.008 (2)
C3	0.059 (2)	0.084 (3)	0.058 (2)	0.014 (2)	0.008 (2)	−0.010 (2)
C4	0.045 (2)	0.081 (3)	0.065 (2)	−0.006 (2)	−0.0058 (19)	−0.018 (2)
O4	0.106 (3)	0.093 (2)	0.101 (3)	−0.003 (2)	0.002 (2)	−0.009 (2)
C5	0.056 (2)	0.054 (2)	0.072 (3)	−0.0070 (17)	−0.0050 (19)	−0.0186 (19)
O5	0.205 (6)	0.134 (4)	0.127 (4)	0.023 (4)	0.005 (4)	0.011 (3)
C6	0.053 (2)	0.100 (4)	0.106 (4)	−0.007 (3)	−0.005 (2)	−0.010 (3)
O6	0.175 (6)	0.205 (6)	0.192 (7)	−0.012 (6)	0.017 (6)	0.012 (5)
C7	0.065 (3)	0.106 (4)	0.085 (3)	−0.002 (3)	−0.012 (3)	0.008 (3)
C8	0.069 (2)	0.060 (2)	0.083 (3)	−0.009 (2)	−0.017 (3)	−0.014 (2)
C9	0.058 (2)	0.062 (2)	0.082 (3)	−0.0060 (18)	−0.002 (2)	−0.017 (2)
C10	0.110 (4)	0.055 (2)	0.083 (3)	0.001 (3)	0.005 (3)	−0.019 (2)
C11	0.121 (5)	0.058 (3)	0.106 (5)	−0.014 (3)	0.013 (4)	−0.009 (3)
C12	0.096 (4)	0.092 (4)	0.130 (5)	−0.024 (3)	0.013 (4)	−0.003 (4)
C13	0.083 (3)	0.066 (3)	0.103 (4)	−0.017 (2)	0.010 (3)	−0.006 (3)
C14	0.138 (5)	0.081 (4)	0.093 (4)	0.020 (4)	−0.023 (4)	−0.014 (3)
C15	0.181 (8)	0.108 (5)	0.089 (5)	0.051 (5)	−0.011 (5)	−0.028 (4)
C16	0.177 (8)	0.101 (5)	0.081 (4)	0.045 (5)	0.025 (5)	−0.007 (4)
C17	0.185 (7)	0.061 (3)	0.084 (4)	−0.018 (4)	0.031 (5)	−0.003 (3)
C18	0.219 (11)	0.135 (7)	0.151 (8)	−0.035 (8)	0.000 (8)	0.040 (6)
C19	0.188 (11)	0.188 (9)	0.213 (12)	0.032 (8)	−0.038 (9)	0.028 (9)
C20	0.104 (4)	0.065 (2)	0.058 (2)	−0.009 (3)	0.006 (2)	−0.011 (2)

### Geometric parameters (Å, °)

**Table:**

S—O3	1.387 (4)	C8—C9	1.542 (6)
S—O4	1.437 (4)	C8—C10	1.550 (7)
S—O2	1.563 (3)	C8—H8A	0.9800
S—O1	1.567 (3)	C9—C13	1.518 (6)
O1—C2	1.407 (6)	C9—H9A	0.9800
C1—C2	1.537 (7)	C10—C11	1.326 (8)
C1—C5	1.541 (6)	C10—C14	1.458 (8)
C1—H1A	0.9700	C11—C17	1.402 (8)
C1—H1B	0.9700	C11—C12	1.515 (9)
O2—C3	1.444 (6)	C12—C13	1.510 (8)
C2—C3	1.551 (6)	C12—H12A	0.9700
C2—H2A	0.9800	C12—H12B	0.9700
C3—C4	1.567 (6)	C13—H13A	0.9700
C3—H3B	0.9800	C13—H13B	0.9700
C4—C6	1.494 (6)	C14—C15	1.536 (10)
C4—C20	1.526 (6)	C14—H14A	0.9300
C4—C5	1.543 (5)	C15—C16	1.258 (11)
C5—C9	1.537 (6)	C15—H15A	0.9300
C5—H5A	0.9800	C16—C17	1.342 (11)
O5—C18	1.300 (10)	C17—H17A	0.9300
O5—C16	1.410 (9)	C18—H18A	0.9700
C6—C7	1.556 (7)	C18—H18B	0.9700
C6—H6A	0.9700	C19—H19A	0.9600
C6—H6B	0.9700	C19—H19B	0.9600
O6—C19	1.339 (11)	C19—H19C	0.9600
O6—C18	1.442 (10)	C20—H20A	0.9600
C7—C8	1.532 (6)	C20—H20B	0.9600
C7—H7A	0.9700	C20—H20C	0.9600
C7—H7B	0.9700		
			
O3—S—O4	119.2 (3)	C9—C8—H8A	106.7
O3—S—O2	108.8 (3)	C10—C8—H8A	106.7
O4—S—O2	109.0 (2)	C13—C9—C5	113.7 (4)
O3—S—O1	111.0 (3)	C13—C9—C8	109.6 (4)
O4—S—O1	109.7 (2)	C5—C9—C8	106.9 (3)
O2—S—O1	96.84 (17)	C13—C9—H9A	108.9
C2—O1—S	110.8 (3)	C5—C9—H9A	108.9
C2—C1—C5	103.2 (3)	C8—C9—H9A	108.9
C2—C1—H1A	111.1	C11—C10—C14	120.7 (6)
C5—C1—H1A	111.1	C11—C10—C8	123.5 (5)
C2—C1—H1B	111.1	C14—C10—C8	115.7 (5)
C5—C1—H1B	111.1	C10—C11—C17	120.2 (7)
H1A—C1—H1B	109.1	C10—C11—C12	122.0 (6)
C3—O2—S	112.7 (3)	C17—C11—C12	117.7 (7)
O1—C2—C1	111.9 (4)	C13—C12—C11	112.9 (5)
O1—C2—C3	105.3 (4)	C13—C12—H12A	109.0
C1—C2—C3	106.1 (4)	C11—C12—H12A	109.0
O1—C2—H2A	111.1	C13—C12—H12B	109.0
C1—C2—H2A	111.1	C11—C12—H12B	109.0
C3—C2—H2A	111.1	H12A—C12—H12B	107.8
O2—C3—C2	105.0 (4)	C12—C13—C9	110.0 (4)
O2—C3—C4	114.0 (4)	C12—C13—H13A	109.7
C2—C3—C4	106.0 (4)	C9—C13—H13A	109.7
O2—C3—H3B	110.5	C12—C13—H13B	109.7
C2—C3—H3B	110.5	C9—C13—H13B	109.7
C4—C3—H3B	110.5	H13A—C13—H13B	108.2
C6—C4—C20	110.6 (4)	C10—C14—C15	113.0 (7)
C6—C4—C5	109.9 (4)	C10—C14—H14A	123.5
C20—C4—C5	114.3 (4)	C15—C14—H14A	123.5
C6—C4—C3	114.7 (4)	C16—C15—C14	122.2 (8)
C20—C4—C3	109.1 (4)	C16—C15—H15A	118.9
C5—C4—C3	97.7 (3)	C14—C15—H15A	118.9
C9—C5—C1	120.4 (4)	C15—C16—C17	121.2 (8)
C9—C5—C4	111.9 (3)	C15—C16—O5	125.8 (10)
C1—C5—C4	105.2 (3)	C17—C16—O5	112.9 (9)
C9—C5—H5A	106.1	C16—C17—C11	122.5 (8)
C1—C5—H5A	106.1	C16—C17—H17A	118.8
C4—C5—H5A	106.1	C11—C17—H17A	118.8
C18—O5—C16	119.0 (8)	O5—C18—O6	96.1 (10)
C4—C6—C7	110.4 (4)	O5—C18—H18A	112.5
C4—C6—H6A	109.6	O6—C18—H18A	112.5
C7—C6—H6A	109.6	O5—C18—H18B	112.5
C4—C6—H6B	109.6	O6—C18—H18B	112.5
C7—C6—H6B	109.6	H18A—C18—H18B	110.1
H6A—C6—H6B	108.1	O6—C19—H19A	109.5
C19—O6—C18	106.0 (9)	O6—C19—H19B	109.5
C8—C7—C6	112.2 (4)	H19A—C19—H19B	109.5
C8—C7—H7A	109.2	O6—C19—H19C	109.5
C6—C7—H7A	109.2	H19A—C19—H19C	109.5
C8—C7—H7B	109.2	H19B—C19—H19C	109.5
C6—C7—H7B	109.2	C4—C20—H20A	109.5
H7A—C7—H7B	107.9	C4—C20—H20B	109.5
C7—C8—C9	110.9 (4)	H20A—C20—H20B	109.5
C7—C8—C10	115.7 (4)	C4—C20—H20C	109.5
C9—C8—C10	109.6 (4)	H20A—C20—H20C	109.5
C7—C8—H8A	106.7	H20B—C20—H20C	109.5
			
O3—S—O1—C2	−84.0 (4)	C6—C7—C8—C10	−178.2 (4)
O4—S—O1—C2	142.3 (3)	C1—C5—C9—C13	−54.0 (5)
O2—S—O1—C2	29.3 (3)	C4—C5—C9—C13	−178.3 (4)
O3—S—O2—C3	98.6 (4)	C1—C5—C9—C8	−175.0 (3)
O4—S—O2—C3	−129.9 (3)	C4—C5—C9—C8	60.7 (4)
O1—S—O2—C3	−16.4 (3)	C7—C8—C9—C13	178.7 (4)
S—O1—C2—C1	−146.1 (3)	C10—C8—C9—C13	49.7 (5)
S—O1—C2—C3	−31.3 (4)	C7—C8—C9—C5	−57.8 (4)
C5—C1—C2—O1	126.6 (4)	C10—C8—C9—C5	173.3 (3)
C5—C1—C2—C3	12.3 (4)	C7—C8—C10—C11	−146.2 (5)
S—O2—C3—C2	0.0 (4)	C9—C8—C10—C11	−19.9 (6)
S—O2—C3—C4	115.6 (3)	C7—C8—C10—C14	37.1 (6)
O1—C2—C3—O2	18.9 (4)	C9—C8—C10—C14	163.4 (4)
C1—C2—C3—O2	137.6 (4)	C14—C10—C11—C17	0.6 (7)
O1—C2—C3—C4	−102.1 (4)	C8—C10—C11—C17	−176.0 (4)
C1—C2—C3—C4	16.6 (5)	C14—C10—C11—C12	179.8 (5)
O2—C3—C4—C6	90.8 (5)	C8—C10—C11—C12	3.3 (8)
C2—C3—C4—C6	−154.2 (4)	C10—C11—C12—C13	−16.9 (8)
O2—C3—C4—C20	−33.9 (5)	C17—C11—C12—C13	162.4 (4)
C2—C3—C4—C20	81.1 (5)	C11—C12—C13—C9	47.5 (6)
O2—C3—C4—C5	−153.0 (4)	C5—C9—C13—C12	175.0 (4)
C2—C3—C4—C5	−38.0 (4)	C8—C9—C13—C12	−65.5 (5)
C2—C1—C5—C9	−165.2 (4)	C11—C10—C14—C15	−2.4 (7)
C2—C1—C5—C4	−37.7 (4)	C8—C10—C14—C15	174.5 (4)
C6—C4—C5—C9	−61.2 (5)	C10—C14—C15—C16	1.9 (9)
C20—C4—C5—C9	63.9 (5)	C14—C15—C16—C17	0.5 (12)
C3—C4—C5—C9	178.9 (3)	C14—C15—C16—O5	178.7 (5)
C6—C4—C5—C1	166.4 (4)	C18—O5—C16—C15	52.1 (13)
C20—C4—C5—C1	−68.6 (4)	C18—O5—C16—C17	−129.6 (9)
C3—C4—C5—C1	46.5 (4)	C15—C16—C17—C11	−2.7 (11)
C20—C4—C6—C7	−71.7 (5)	O5—C16—C17—C11	179.0 (5)
C5—C4—C6—C7	55.5 (5)	C10—C11—C17—C16	2.1 (8)
C3—C4—C6—C7	164.4 (4)	C12—C11—C17—C16	−177.2 (6)
C4—C6—C7—C8	−54.6 (6)	C16—O5—C18—O6	−167.1 (8)
C6—C7—C8—C9	56.2 (6)	C19—O6—C18—O5	130.1 (10)

### Hydrogen-bond geometry (Å, °)

**Table:**

*D*—H···*A*	*D*—H	H···*A*	*D*···*A*	*D*—H···*A*
C20—H20A···O3^i^	0.96	2.50	3.338 (5)	146

Symmetry codes: (i) *x*, *y*, *z*−1.
